# Eating disorders in young males: moving beyond stereotype and stigma

**DOI:** 10.1186/2050-2974-2-S1-O54

**Published:** 2014-11-24

**Authors:** Chloe Shu, Karina Limburg, Hunna Watson, Chris Harris, Julie McCormack, Kimberley Hoiles, David Forbes

**Affiliations:** 1 Eating Disorders Program, Specialised Child and Adolescent Mental Health Service, Child and Adolescent Health Service, Department of Health in Western Australia, Perth, Australia; 2 UNC Center of Excellence for Eating Disorders, University of North Carolina, Chapel Hill, North Carolina, USA; 3 YouthFocus, Perth, Australia; 4 School of Paediatrics and Child Health, Faculty of Medicine, Dentistry and Health Sciences, The University of Western Australia, Perth, Australia

## Objective

To provide knowledge about the clinical presentation of eating disorders in young males (< 18 years).

## Method

The sample comprised young males with eating disorders (n = 53) and females with eating disorders (n = 704). The data source was the Helping to Outline Paediatric Eating Disorders (HOPE) Project registry (N ~ 1000), a prospective and ongoing registry study comprising consecutive paediatric tertiary eating disorder referrals.

## Results

Young males with eating disorders more commonly presented with unspecified eating disorders (40%). In comparison to young females with eating disorders young males were less likely to report self-induced purging, endorsed lower weight concern, and presented with an earlier age of onset. Young males and females presented with a similar duration of untreated illness.

## Discussion

Young males with eating disorders are an understudied group who are systematically different from young females with eating disorders. Diagnostic classification, assessment instruments, conceptualisation and treatment methods need to be refined to improve application to young males.

This abstract was presented in the **Service Initiatives: Child and Adolescent** stream of the 2014 ANZAED Conference.

